# Association between Sickle Cell Trait and the Prevalence and Severity of Diabetic Retinopathy

**DOI:** 10.1371/journal.pone.0159215

**Published:** 2016-07-14

**Authors:** Majed Al Harbi, Rajiv Khandekar, Igor Kozak, Patrik Schatz

**Affiliations:** 1 Vitreoretinal Division, King Khaled Eye Specialist Hospital, Riyadh, Kingdom of Saudi Arabia; 2 Department of Ophthalmology, Clinical Sciences, Skane County University Hospital, Lund University, Lund, Sweden; Boston University School of Medicine, UNITED STATES

## Abstract

**Purpose:**

To determine whether Sickle cell trait (SCT) is associated with an increased severity of diabetic retinopathy.

**Methods:**

This was a single center retrospective study case control study of 100 eyes of 100 patients with diabetes mellitus (DM) with SCT (SCT group) and 100 eyes of 100 age-matched patients with DM without SCT (control group). The main outcome measure was the difference in the prevalence of sight threatening DR [here defined as diabetic macular edema (DME) and/or proliferative diabetic retinopathy (PDR)], between the SCT and control groups. Secondary outcome measures included differences in visual acuity, ocular comorbidities, intraocular pressure, glycemic control as assessed by random blood glucose measurement, diabetes duration, nephropathy, hyperlipidemia and hypertension.

**Results:**

The SCT group had statistically significantly shorter duration of DM (median [25% quartile] 15 [8.3] years versus 20 [14.7] years, respectively)(*P*<0.001) and presented with statistically better metabolic control (mean difference 1.6 mmol/l, (95% confidence interval [CI], 0.1–3.3;*P* = 0.03). The prevalence of PDR and/or DME was significantly lower in the SCT group (58%) compared to the control group, (95%)(*P*<0.001). The absence of SCT (adjusted odds ratio [AOR] = 24; 95% CI, 8–72; *P*<0.001) and longer duration of DM (AOR = 1.1 [95% CI, 1.02–1.13]; *P* = 0.003) were independent predictors of PDR and/or DME.

**Conclusions:**

SCT seems to protect against the development and progression of DR. This may have implications for monitoring and screening. Prospective studies are required to confirm this association. If true, this association may indicate an increased blood glucose buffering capacity of abnormal hemoglobin.

## Introduction

Metabolic control and duration of diabetes mellitus (DM) are major factors affecting the risk of microvascular complications, including diabetic retinopathy (DR) in type 1 and type 2 DM.[1–6] However, despite poor metabolic control, DR takes a long time to develop in some patients, whereas in others DR develops despite good metabolic control. Zhang et al. reported that DR developed in about 10% of type 1 diabetes patients under good metabolic control, whereas 40% of type 1 diabetes patients remained free of DR despite poor metabolic control.[7] Other factors such as body mass index (BMI), fluctuations in short term blood glucose level and genetic factors may affect the risk of developing DR and the progression of DR.[8–10]

Sickle cell disorder (SCD) results from a homozygous single amino acid substitution of valine for glutamic acid being in position 6 of the beta globin chain of hemoglobin. This substitution results in a tendency for hemoglobin polymerization, sickling of erythrocytes and ischemic complications in various organs, significantly increasing the risk of mortality.[11] Sickle cell trait (SCT) is the heterozygous form that is generally considered relatively benign. However both SCD and SCT may lead to retinopathy and proliferative retinopathy.[12,13] SCT is much more common in the population than SCD.[14] Hence, a significant number of people may have concurrent DM and SCT, especially type 2 DM which is increasing world-wide.[14]

A recent study found increased oxidative stress, abnormal blood rheology and vascular dysfunction in patients with concurrent DM and SCT.[14] Thus, the coexisting SCT and DM may have additive effects on the development of microvascular complications. A retrospective study of 821 African American patients with diabetes (110 of whom had SCT) reported that SCT did not increase the risk of microvascular complications, including retinopathy.[15] Interestingly, SCT participants had significantly lower prevalence of retinopathy, peripheral vascular disease, and end-stage kidney disease. After adjustment for diabetes duration, age, insulin use, and gender, the differences in the prevalence of microvascular complications were no longer observed.[15] However study data were obtained by telephone interview and the degree of retinopathy was not assessed.[15]

The purpose of this study was to assess the association between SCT and the severity of DR among Saudi patients with diabetes at a tertiary eye hospital.

## Methods

This study was approved by the IRB at King Khaled Eye Specialist Hospital. The investigation was conducted according to the principles expressed in the Declaration of Helsinki. The data was analyzed retrospectively and anonymously, thus no consent was obtained. This was a retrospective study of 100 consecutive, diabetic patients admitted to the King Khaled Eye Specialist Hospital (KKESH; Riyadh, Saudi Arabia) diagnosed with SCT (SCT group) between 2010–2013. SCT was diagnosed as part of routine work-up during admission at KKESH with the SAS™ Sickle Cell Test (Modified Nalbadian; SA Scientific Ltd., San Antonio, TX, USA), and was verified by hemoglobin electrophoresis. Diabetic patients without SCT who presented over the same period were randomly selected to comprise an age-matched control group. Both groups were initially identified based on diagnostic coding however it was subsequently verified that all controls were negative for the sickle cell test and that all patients were positive for the test.

Diabetes in patients and controls was defined as a positive history of either type 1 or type 2 diabetes. Random blood glucose measurement was obtained in all patients and controls. All patients and controls were seen and examined by a retina specialist at King Khaled Eye Specialist Hospital. As the system for grading of retinopathy was not pre-specified and data was retrieved retrospectively, we graded retinopathy as either proliferative or non-proliferative, as noted in the patients´ files by the examining ophthalmologist.

Previous data indicate that the prevalence of diabetes retinopathy in the Saudi diabetic population is at least 30%.[16–18] Considering that this study was performed in the setting of a tertiary ophthalmic referral center where many patients are referred for management of ophthalmic complications of diabetes, we tentatively assumed (for the purpose of power calculation) that the rate of proliferative DR (PDR) in diabetic patients without SCT would be 65%, and in diabetic patients with SCT it would be 85%. To achieve a 5% level of significance and 90% power in a retrospective cohort study with two arms, at least 95 cases were required in each arm. To compensate for patient dropout, we included 100 cases of DM with SCT (SCT group) and 100 DM cases without SC as the control group. Openepi software was used to calculate the sample size. (Dean AG, Sullivan KM, Soe MM. OpenEpi: Open Source Epidemiologic Statistics for Public Health, Version. www.OpenEpi.com, updated 2015/05/04, accessed 2015/12/20.)

The main outcome measure was pre-defined as difference in the rate (prevalence) between groups of sight threatening retinopathy (here defined as PDR and/or diabetic macular edema (DME), the latter requiring treatment by either laser or intravitreal injection). Secondary outcome measures included differences in the prevalence (a positive or negative history) of other ocular disorders, ocular surgeries, glycemic control (defined as level of random blood glucose measurement), diabetes duration, nephropathy (defined as history of nephropathy or on dialysis or renal transplant), hyperlipidemia (defined as requiring medication) and hypertension (defined as requiring medication).

To minimize bias, the patients in the control group were age-matched to the SCT group. For calculations of between-group difference in DR and differences in general ocular status, right eyes of patients and controls were compared, in order to minimize any potential bias from possible pairwise correlation of findings between the 2 eyes of a given patient in either group.

Data was collected on a pre-specified data collection form and transferred to Access® spreadsheet (Microsoft Corp., Redmond, WA, USA). Statistical Package for the Social Sciences (SPSS version 16) (IBM Corp., Armonk, NY, USA) was used for statistical analysis. Univariate and multivariate analyses were performed with both parametric and non-parametric methods. For normally distributed quantitative variables, the mean and the standard deviations were calculated. For variables with non-normal distribution, the median, 25% quartile, minimum and maximum values were calculated. Difference of means was estimated and 95% confidence interval (CI) and two-sided *P* values were calculated for comparison of the study outcomes between groups. To compare the results of nonparametric variables between groups, the Kruskal Wallis (K-W) test was used with two-sided *P* values. For qualitative variables, the frequencies and percentage proportions were calculated. A subgroup comparison was performed by calculating the relative risks, 95% CIs and two-sided *P* values. For more than 2 dependent variables, the chi-square value and two-sided *P* values were calculated. To identify the interaction of different variables while associating them to the outcomes, bi-nominal regression analysis using the step-out method was used. Variables significantly associated to the outcome were included in the model and then removed from the model if not statistically significant.

## Results

There were 100 patients in each group (SCT group and control group). The age and gender of the two groups were similar (Table 1).

**Table 1:** Demographics of patients with diabetes mellitus with and without sickle cell trait.

	SCT	Controls	Validation
Age. Mean (sdv)	61.5 (14.4)	62.7 (11.4)	*P* = 0.5
Gender. Male, female (numbers)	57, 43	65, 35	OR = 0.7, 95% CI: 0.4–1.3, *P* = 0.2

SCT = Sickle cell trait, SCT group had diabetes mellitus with SCT, Control group of patients had diabetes mellitus without SCT, OR = odds ratio CI = confidence interval, Sdv = Standard deviation.

The duration of DM, other systemic complications and risk factors for DR in both groups are presented in Table 2. The majority of patients in both groups had type 2 DM (Table 2). The mean duration of DM in the SCT group was statistically significantly shorter at 15 (8.3) years compared to 20 (14.7) years for the control group (*P*<0.001). The SCT group presented with statistically significantly better metabolic control (mean difference 1.6 mmol/l, 95% CI, 0.1–3.3; *P* = 0.03).

**Table 2:** Comparison of systemic variables in patients with diabetes mellitus with and without sickle cell trait (SCT).

	SCT (n = 100)	Controls (n = 100)	Validation
Duration (years) of DM. Median, 25% quartile, range	15, 8.3, 6–45	20, 14.7, 1–40	K-W *P*<0.001
Random Blood glucose. Mean, sdv (mmol/l)	12.3, 4.9	13.8, 5.4	Mean diff = 1.6; 95% CI: 0.1–3.3, *P* = 0.03
Type of DM. Type 1 DM, Type 2 DM, (numbers)	7, 93	6, 94	*P* = 0.8
Hypertension. Present, absent (numbers)	78, 22	70, 30	OR = 1.5, 95% CI: 0.8–2.9; *P* = 0.2
Nephropathy. Present, absent (numbers)	20, 80	15, 85	OR = 1.4, 95% CI: 0.7–2.9, *P* = 0.4
Hyperlipidemia. Present, absent, N/A (numbers)	33, 30, 37	34, 46, 20	OR = 1.4, 95% CI: 0.8–2.9, *P* = 0.2
Random blood glucose. Less or equal to 10 mmol/l, >10 mmol/l (numbers)	36, 64	17, 83	OR = 2.7, 95% CI: 1.4–5.3, *P*<0.01

SCT = Sickle cell trait, DM = diabetes mellitus, SCT group had DM with SCT, Control group of patients had DM without SCT, mmol/l = millimoles per liter SCT = Sickle cell trait, DM = diabetes mellitus, Sdv = standard deviation, OR = odds ratio, CI = confidence interval, K-W = Kruskal Wallis test, N/A = not available.

To compare the ocular profile and past ocular surgeries, we included the right eyes only of each participant in both groups (Table 3). Ocular comorbidity, visual acuity and intraocular pressure in both groups were similar. A history of cataract surgery was statistically significantly higher in the SCT group (N = 67 eyes vs. N = 42 eyes; *P*<0.001).

**Table 3:** Ocular status of right eyes of patients with diabetes mellitus with and without sickle cell trait.

	SCT (n = 100)	Controls (n = 100)	Validation
Lid /lacrimal apparatus	2	1	
Corneal pathology	5	6	
Glaucoma	16	8	
Cataract	93	97	
Ophthalmoplegia	0	0	
Optic neuritis	2	0	
Other	0	2	
Best corrected visual acuity. 20/60 to 20/20, 20/200 to <20/60, 20/400 to <20/200, <20/400	51, 21, 12, 16	48, 32, 13, 7	Chi square = 0.8, DF = 3, *P* = 0.4
IOP. Less than or equal to 22 mmHg, >22 mmHg	91, 9	90, 10	OR = 1.1 95% CI: 0.4–2.9, *P* = 0.8
Cataract surgery in past	67	42	*P*<0.001
Glaucoma surgery in past	0	0	
Other eye surgery	14	13	
No eye surgery in past	19	45	

SCT = Sickle cell trait, DM = diabetes mellitus, SCT group had DM with SCT, Control group of patients had DM without SCT, DF = degrees of freedom, OR = odds ratio, CI = confidence interval, Diff = difference, *P*<0.05 is statistically significant.

The prevalence of PDR and related eye complications in the right eyes of both groups are presented in Table 4. The prevalence of PDR and/or DME was statistically significantly lower in the SCT group compared to the control group (58%, vs 95% respectively, *P*<0.001, Χ^2^ = 49, Degrees of freedom = 3).

**Table 4:** Association between sickle cell trait and the prevalence of proliferative diabetic retinopathy, its complications and/or diabetic macular edema.

	SCT group (n = 100 eyes)	Controls (n = 100 eyes)	Validation
Proliferative Diabetic Retinopathy. Present, absent	50, 50	96, 4	OR = 0.04, 95% CI: 01–0.1, *P*<0.001
Diabetic macular edema. Present, absent	28, 72	54, 46	OR = 0.3, 95% CI: 0.2–0.6, P<0.001
Neovascular glaucoma. Present, absent	4, 96	9, 91	OR = 0.4, 95% CI: 0.1–1.4, *P* = 0.2
Vitreous haemorrhage. Present, absent	22, 78	39, 61	OR = 0.4, 95% CI: 0.2–0.8, *P* = 0.009
Traction retinal detachment. Present, absent	26, 74	11, 89	OR = 2.8, 95% CI: 1.3–6.1, *P* = 0.006
Pan retinal photocoagulation in past. Yes, no	50, 50	97, 3	OR = 0.03, 95% CI: 0.01–0.1, *P*<0.001
Focal laser in past. Yes, no	14, 86	36, 64	OR = 0.3, 95% CI: 0.1–0.6, *P*<0.001
Intravitreal injection of anti-vascular growth factor. Yes, no	19, 81	34, 66	OR = 0.5, 95% CI: 0.2–0.9, *P*<0.001

NVG = Neovascular glaucoma, SCT = Sickle cell trait, SCT group had diabetes mellitus with SCT, Control group of patients had diabetes mellitus without SCT, DF = degrees of freedom, OR = odds ratio, CI = confidence interval, Diff = difference, *P<0*.*05* is statistically significant.

The association of SCT to the presence of PDR and/or DME in presence of other known risk factors of progression of DR is presented in (Table 5). Logistic regression analysis indicated that the absence of SCT (Adjusted Odds Ratio [AOR] = 24 with 95% CI, 8–72; *P*<0.001) and longer duration of DM (AOR = 1.1 with 95% CI, 1.02–1.13; P = 0.003) were independent predictors of PDR and/or DME, whereas lack of hypertension (AOR = 0.5 with 95% CI, 0.2–0.9; *P* = 0.02) and lack of diabetic nephropathy (AOR = 0.2 with 95% CI = 0.1–0.5, *P*<0.001) were independent protectors. Of these parameters, the largest effect on the risk of PDR and/or DME was produced by the absence of SCT. Although controls had poorer glycemic control than patients with SCT (Table 2), glycemic control was not a significant independent risk factor (*P* = 0.4) for PDR and/or DME in the logistic regression analysis presented in Table 5.

**Table 5:** Interaction of significant risk factors on association between sickle cell trait versus proliferative diabetic retinopathy and/or diabetic macular edema, based on logistic regression analysis.

	Adjusted OR	95% CI	*P* value
SCT. Present, absent	1, 24	8–72	<0.001
Duration of DM	1.1	1.02–1.13	0.003
Hypertension. Present, absent	1, 0.5	0.2–0.9	0.02
Diabetic nephropathy. Present, absent	1, 0.2	0.1–0.5	<0.001

SCT = Sickle cell trait, DM = diabetes mellitus, CI = confidence interval, Diff = difference, OR = odds ratio, *P<0*.*05* is statistically significant.

## Discussion

SCD and SCT are relatively common in individuals whose ancestors lived in sub-tropical regions where malaria is prevalent. SCT in malaria endemic area confers a selective advantage, leading to attenuated symptoms during malaria infection.[19] In Saudi Arabia, approximately 4.2% of the population carries SCT and 0.26% has SCD. The highest prevalence is in the Eastern province of the country where approximately 17% of the population has SCT and 1.2% has SCD.[20]

The outcomes of our study indicate that SCT may be protective not only against the manifestation of malaria, but also against the development, progression and complications of diabetic retinopathy. This observation was made for neovascular disease and for DME. These 2 conditions are both driven by ischemia and vascular endothelial growth factor (VEGF) (over)production and are responsive to intravitreal anti-VEGF injections.[21]

The apparent protective effect of SCT on the severity of DR was confirmed in the present study even when data was adjusted for duration and glycemia (defined as random blood glucose), which are two major factors affecting the progression of DR (Table 5). Although controls had poorer glycemic control than patients with SCT (Table 2), glycemic control was not a significant independent risk factor (*P* = 0.4) for PDR and/or DME in the logistic regression analysis presented in Table 5. Additionally, there were no differences between groups in the rate of nephropathy, hypertension or hyperlipidemia which are considered potential risk factors for development and progression of DR (Tables 1 and 2).[1–10] Various indicators (such as vitreous hemorrhage, previous panretinal photocoagulation and or focal laser) of the 2 most significant vision-threatening complications of DR, PDR and DME, were significantly lower in the SCT group (Table 4). Additionally, regression analysis of the risk factors for an association between sickle cell trait (SCT) PDR and/or DME indicate that SCT is the strongest protector of the severity of DR compared to other risk factors such as duration, hypertension and nephropathy (Table 5). Other ocular comorbidities and visual acuity did not differ between groups.

The reason for any potential protective effect of SCT on the severity of DR remains ambiguous, however the observation concurs to some extent with a previous report.[15] In that study, after adjustment for diabetes duration, age, insulin use, and gender, the differences in the prevalence of microvascular complications were no longer observed. On the other hand, in this study, age and gender of the two groups were similar (Table 1), whereas diabetes duration was longer and metabolic control (defined as random blood glucose) poorer in the control group. However, regression analysis indicated that the lack of SCT conferred a higher risk of PDR, compared to either hypertension, nephropathy or diabetes duration (Table 5).

Protective genetic factors have been documented in other ophthalmic diseases such as, age related macular degeneration and thyroid associated orbitopathy.[22,23] Additionally, there is evidence from an in vitro study that abnormal hemoglobins may act as a buffer and absorb large amounts of blood glucose, potentially preventing hyperglycemia-induced tissue damage.[24] Another possibility is that glucose-bound abnormal hemoglobins have different biological properties, being less stable than normal hemoglobin upon condensation with glucose,[24] inducing some biological events that protects against the development and progression of DR. This may indicate a possibility for novel blood glucose buffering approaches as pharmacotherapy for DM.

There are some limitations to this study. The subjects in both groups were recruited from patients admitted to a tertiary ophthalmic care center. Hence, this cohort may not be representative of population of sickle cell patients and diabetic patients in general. For example, the majority of the control group had PDR (Table 4). Therefore, there may be some referral bias in the current study. Additional sources of bias include the lack of detailed information regarding metabolic control, such as HbA1C levels. Landmark studies, such as the Diabetes Control and Complications Trial (DCCT) and UK Prospective Diabetes Study (UKPDS), found intensive glycemic control was effective in reducing the rate of DR progression in both type 1 and type 2 DM.[1, 4–6] However due to the retrospective design and the fact that random blood glucose, but not HbA1C, is used routinely in our hospital in the evaluation of diabetes patients, we were not able to evaluate this parameter in the study. Thus, although random blood glucose was not a significant risk factor for PDR and/or DME, we cannot rule out that the observed protective effect of SCT on the level of diabetic retinopathy was in fact due to better metabolic control in this group.

We did not assess the level of several risk factors, such as nephropathy or hypertension, merely their presence or absence. On the other hand, it is worth to note that all the patients were diagnosed with SCT as part of hospital routine work up, and that SCT on its own is not a reason for ophthalmology referral in Saudi Arabia. This means that there was no facilitated referral for these patients, and therefore such a potential source of bias should not have been present here. Nevertheless, the frequency of cataract surgery in SCT patients was about 25% higher than that of the controls (Table 3), potentially indicating more intensive exposure to general health and eye care in this group.

In conclusion, SCT may be protective against the development and progression of diabetic retinopathy. This potential association requires further investigations in prospective studies, such as matched case-control study, or a longitudinal study, which should include measurement of HbA1C levels. A protective effect of SCT in DR may have a significant impact in the management of DR and screening intervals in countries with a high prevalence of SCT and DM. If the protective effect of SCT is verified, the potential exists for novel blood glucose buffering approaches for pharmacotherapy for DM.
